# How the New European Union’s (Pictorial) Tobacco Health Warnings Influence Quit Attempts and Smoking Cessation: Findings from the 2016–2017 International Tobacco Control (ITC) Netherlands Surveys

**DOI:** 10.3390/ijerph16214260

**Published:** 2019-11-02

**Authors:** Dirk-Jan A. van Mourik, Math J. J. M. Candel, Gera E. Nagelhout, Marc C. Willemsen, Hua-Hie Yong, Bas van den Putte, Geoffrey T. Fong, Hein de Vries

**Affiliations:** 1 Department of Health Promotion, Maastricht University (CAPHRI), 6229 HA Maastricht, Limburg, The Netherlands; gera.nagelhout@maastrichtuniversity.nl (G.E.N.); marc.willemsen@maastrichtuniversity.nl (M.C.W.); hein.devries@maastrichtuniversity.nl (H.d.V.); 2 Department of Methodology and Statistics, Maastricht University (CAPHRI), 6229 HA Maastricht, Limburg, The Netherlands; math.candel@maastrichtuniversity.nl; 3 Department of Family Medicine, Maastricht University (CAPHRI), 6229 HA Maastricht, Limburg, The Netherlands; 4 IVO Research Institute, 2595 AA The Hague, Zuid Holland, The Netherlands; 5 Netherlands Expertise Center for Tobacco Control (NET), Trimbos Institute, 3521 VS Utrecht, The Netherlands; 6 School of Psychology, Deakin University, Geelong 3220, Australia; hua.yong@deakin.edu.au; 7 Department of Communication, University of Amsterdam (ASCoR), 1001 NG Amsterdam, Noord Holland, The Netherlands; S.J.H.M.vandenPutte@uva.nl; 8 Department of Psychology, University of Waterloo, Waterloo, ON N2L 3G1, Canada; geoffrey.fong@uwaterloo.ca; 9 School of Public Health and Health Systems, University of Waterloo, Waterloo, ON N2L 3G1, Canada; 10 Ontario Institute for Cancer Research, Toronto, ON M5G 0A3, Canada

**Keywords:** pictorial health warnings, smokers, quit attempts, smoking cessation, structural equation modeling

## Abstract

In 2016, the Netherlands was required to introduce new European Union (EU)’s (pictorial) tobacco health warnings. Our objective was to describe the pathways through which the new EU tobacco health warnings may influence quit attempts and smoking cessation among Dutch smokers. Longitudinal data from 2016 and 2017 from the International Tobacco Control (ITC) Netherlands Survey were used. Smokers who participated in both surveys were included (*N* = 1017). Structural equation modeling was applied to examine the hypothesized pathways. Health warning salience was positively associated with more health worries (β = 0.301, *p* < 0.001) and a more positive attitude towards quitting (β = 0.180, *p* < 0.001), which, in turn, were associated with a stronger quit intention (health worries: β = 0.304, *p* < 0.001; attitude: β = 0.340, *p* < 0.001). Quit intention was a strong predictor of quit attempts (β = 0.336, *p* = 0.001). Health warning salience was also associated with stronger perceived social norms towards quitting (β = 0.166, *p* < 0.001), which directly predicted quit attempts (β = 0.141, *p* = 0.048). Quit attempts were positively associated with smoking cessation (β = 0.453, *p* = 0.043). Based on these findings, we posit that the effect of the EU’s tobacco health warnings on quit attempts and smoking cessation is mediated by increased health worries and a more positive attitude and perceived social norms towards quitting. Making tobacco health warnings more salient (e.g., by using plain packaging) may increase their potential to stimulate quitting among smokers.

## 1. Introduction

In the European Union (EU), pictorial health warnings (PHWs) on the packet of tobacco products [1] were introduced as of May 2016 with the new Tobacco Products Directive [2]. The PHWs cover 65% of the front and back of the packet and are accompanied with a matching textual health warning (THW) and two general THWs on 50% of the lateral sides of the packet [1,2]. Tobacco health warnings are generally used to inform—current or possible future—consumers about the health risks of smoking [3]. Previous studies have suggested that introducing PHWs leads to a greater likelihood of smokers quitting [4,5], but the health warnings evaluated in these studies differed from the EU PHWs in terms of accompanying THWs and graphic portrayals [1,6]. This study aims to examine if, and through which pathways, the new EU tobacco health warnings affect quit attempts and smoking cessation. Our findings may result in a better understanding of the working mechanisms of current EU tobacco health warnings, leading to recommendations about its future use [7,8,9] and accompanying tobacco control policies.

One previous study, with smokers from four high-income countries, examined a mediational model of the impact of tobacco health warnings on subsequent quitting behavior [10]. It revealed that the health warnings were associated with an increase in the extent to which health warnings motivated smokers to think about the health risks of smoking. Thinking led to more worries about the health risks of smoking, which was associated with a stronger quit intention, a strong predictor for attempting to quit. However, there might be alternative pathways of how tobacco health warnings exert effects on quitting behavior [10], and no study has examined the working mechanisms of the new EU tobacco health warnings.

**Figure 1:**
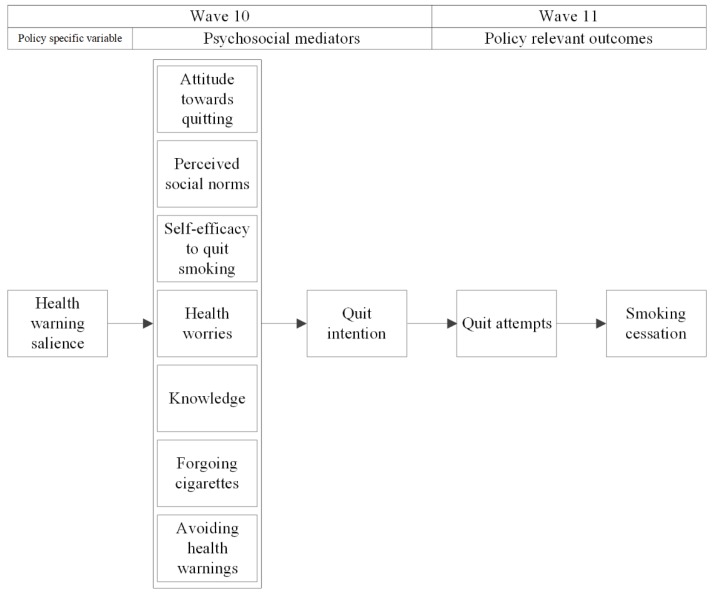
Hypothesized model of the mediational pathways through which the new EU tobacco health warnings on the packet of tobacco products may influence quit attempts and smoking cessation.

The starting point for our mediational model (Figure 1) of the impact of the new EU tobacco health warnings is the International Tobacco Control (ITC) conceptual model of the impact of tobacco control policies [11]. According to the ITC conceptual model, tobacco control policies such as tobacco health warnings have an influence on policy-relevant outcomes (such as quit attempts and smoking cessation) via psychosocial mediators. The ITC conceptual model expects most immediate effects on ‘policy specific variables’. For our mediational model, the policy-specific variable of interest is the salience of the EU tobacco health warnings.

Health warning salience is hypothesized to influence quit intention and behavior through a set of psychosocial mediators [11]. We based the selection of the psychosocial mediators of our mediational model on the content of the health warnings. Attitude towards quitting was selected as the health warnings may influence this (e.g., by pointing out the health risk of smoking). Perceived social norms towards quitting were chosen as a THW addressed this (‘Quit now—stay alive for close family and friends’), but also other health warnings may influence this. Self-efficacy was selected as a THW on each packet aim to increase self-efficacy to quit smoking (‘Quit now! Go to www.ikstopnu.nl., or call the quitline on 0800–1995 (free)). The EU tobacco health warnings may also influence health worries—the health warnings comprise messages that may be perceived as distressing (the THWs point out the health risks of smoking and some PHWs contain strong graphic imagery). Furthermore, knowledge about the health risks of smoking was selected as the health warnings comprise such information [1]. Socio-cognitive models of behavior change [12,13], and previous studies [14,15,16,17,18,19,20] predict that these outcomes are positively associated with quit intention. Quit intention, in turn, is hypothesized to promote quit attempts [15], possibly leading to smoking cessation (policy-relevant outcomes). Furthermore, we added forgoing a cigarette due to health warnings and avoiding of health warnings as two additional behavioral mediators, given that previous studies have shown that PHWs may directly influence these outcomes [21,22,23,24]. Based on previous research, forgoing is hypothesized to influence quit intention positively [10,25], while no impact of avoiding quit intention is expected [25,26].

In sum, the current study aims to examine (1) the impact of the new EU tobacco health warnings on psychosocial mediators and policy-relevant behavioral outcomes, and (2) how these outcomes are interrelated. We will do this by testing a hypothesized mediational model of the pathways through which the EU tobacco health warnings may influence smoking cessation.

## 2. Materials and Methods

### 2.1. Sample

Surveys were conducted via the internet by the research firm Kantar Public. They used a probability-based web database to obtain a sample representative of Dutch smokers aged 15 years and older [27]. Respondents earned points which they could exchange for gift certificates.

We used longitudinal data from the ITC Netherlands Wave 10 (November to December 2016; shortly after introducing the EU tobacco health warnings in May 2016; *N* = 1696) and Wave 11 (November to December 2017; when the health warnings were fully implemented; *N* = 1695) surveys. The total sample population was *N* = 2020. The ITC Netherlands Surveys received ethics clearance from the University of Waterloo’s Office of Research Ethics (ORE # 18920). Respondents were categorized as a smoker if at Wave 10 they smoked at least once a month, and if they had smoked at least 100 cigarettes in their lifetime [28]. From the 1263 smokers from Wave 10, 19.5% was lost to attrition in Wave 11. This resulted in 1017 smokers who participated in both survey waves and who were, therefore, available for the analysis by structural equation modeling.

### 2.2. Measures

Health warning salience and psychosocial mediators were assessed at Wave 10, while we used policy-relevant outcomes from Wave 11. Quit attempts were assessed at Wave 11 by asking smokers from Wave 10 whether they have made any quit attempts since the last survey, and smoking cessation was determined based on their reported smoking status at Wave 11. We assessed smoking status by first asking whether the respondent smoked at least once a day (yes/no). If not, they were asked whether they smoked at least once a week (yes/no). If respondents did not smoke at least once a week, they were asked whether they smoked at least once a month (yes/no). Table 1 describes the constructs and how they were measured in the survey. In the analysis by structural equation modeling, the mean scores were used for health warning salience (Cronbach’s Alpha (α) = 0.793), and self-efficacy (α = 0.748), whereas attitude towards quitting (α = 0.824) was implemented as a latent variable in the analysis by structural equation modeling (only possible when a construct is measured by at least three items). Because we considered the items on knowledge to be formative indicators [29], a sum score (as we considered this as an index score) was calculated for this construct (α = 0.833).

Control variables were gender, age, educational level, level of nicotine dependence, the number of times a respondent participated in the ITC Netherlands cohort (time in the sample), and daily versus non-daily smoking status, as these variables may influence dependent variables and correlate with possible predictors in the path model.

Education was divided into three categories: (1) low (primary education and lower pre-vocational secondary education), (2) moderate (middle pre-vocational secondary education and secondary vocational education), and (3) high (senior general secondary education, (pre-) university education, and higher professional education). The level of nicotine dependence was assessed using the Heaviness of Smoking Index (HSI). The HSI is the sum of two categorical measures—the number of cigarettes smoked per day (four categories: 0–10, 11–20, 21–30, 31+) and the time before smoking the first cigarette of the day (four categories: 61 + minutes, 31–60 min, 6–30 min, 5 min or less). The HSI ranges from 0 to 6, with a higher score indicating a stronger nicotine dependence [30]. We controlled for time in sample as this may influence responses [31]. All control variables were derived from Wave 10.

### 2.3. Statistical Analyses

SPSS version 24 was used to perform attrition, reliability, and correlation analyses, and to examine sample characteristics. All analyses were weighted by gender and age to be representative of the smoker population in the Netherlands. MPlus version 7 was used to examine the hypothesized mediational model by performing structural equation modeling. Although we postulated the effects of salience to be mediated by psychosocial variables according to the model in Figure 1, also direct effects of variables were included, in order to estimate the mediated paths as unbiasedly as possible. In case also direct effects turned out to be significant, these will also be reported.

We used the fully conditional specification method (with the sequential regression procedure) to impute missing data [32]. Several simulation studies suggest that this imputation method produces unbiased parameter estimates and standard errors [33,34]. The number of imputations, 50, was taken to be at least as large as the percentage of cases that had incomplete data due to respondents filling in “Don’t know” as the answer to at least one of the variables (30.8%) [35]. A pathway between warning salience on the one hand and quit attempts or smoking cessation on the other was declared significant if each of the intermediate relations, as depicted in the mediational model in Figure 1, was significant. This so-called Joint Significance Test (JST) for mediation has been shown to provide the best balance between the type I error rate and statistical power [36].

Although analysis with multiply imputed data is valid under the assumption of missingness at random [32], this assumption cannot be tested and requires that the imputation model is correct and thus leads to unbiased imputations. For this reason, a sensitivity analysis was performed using only complete cases. Although such a complete case analysis is valid under the stronger assumption of missingness completely at random, it is also valid in some cases of the weaker assumption of missingness not at random [37]. In this analysis, bootstrapping was used to test the significance of complete mediational pathways between health warning salience on the one hand and smoking cessation on the other in order to replicate the findings from the JST.

To assess model fit, the Comparative Fit Index (CFI), Tucker–Lewis–Index (TLI), and Root–Mean–Square Error of Approximation (RMSEA) was used. An acceptable model fit is indicated by a CFI and TLI above 0.90, and an RMSEA below 0.05 [38].

## 3. Results

### 3.1. Attrition Analyses

Respondents who were lost to attrition in Wave 11 were significantly younger (t = 2.908, *p* = 0.004), had higher self-efficacy (t = −2.515, *p* = 0.012), and reported higher levels of quit intention (t = −2.658, *p* = 0.008) than smokers who remained in the sample.

### 3.2. Sample Characteristics

The sample characteristics of all eligible smokers are shown in Table 2. Twenty percent of the smokers avoided health warnings, while 9% indicated that health warnings stopped them from having a cigarette. Also, 27% attempted to quit smoking, and 10% actually quit smoking.

### 3.3. Correlations

Table 3 displays Spearman correlations between all variables of the mediational model. Health warning salience was positively associated with all psychosocial mediators, except for self-efficacy. Also, most psychosocial mediators were correlated with other psychosocial mediators. These variables were included in the mediational model, as depicted in Figure 1.

### 3.4. Structural Equation Model

**Figure 2:**
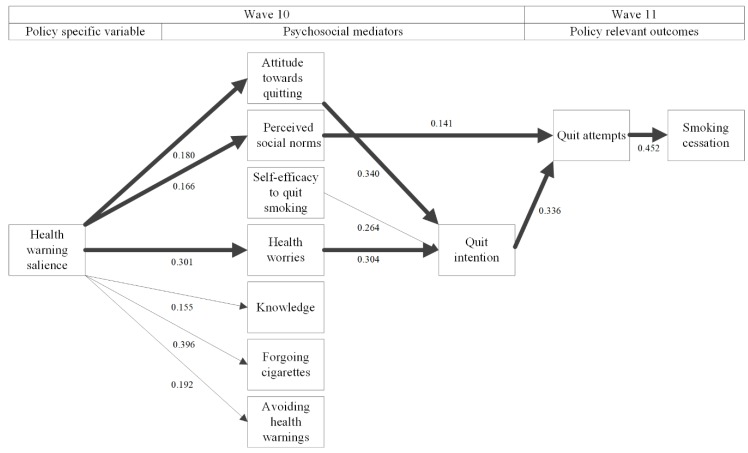
Structural equation model with standardized regression coefficients for the analysis in multiply imputed datasets. Thick lines represent significant pathways between health warning salience and smoking cessation; thin lines represent significant associations that are not part of a significant mediational pathway; no lines between variables denote non-significant associations between them. All psychosocial mediators that are in the second column from the left were allowed to covary.

Table 4 displays the standardized regression coefficients of the structural equation model, while Figure 2 visualizes it. The model fitted the data very well (CFI = 0.992, TLI = 0.961, RMSEA = 0.033), and explained 18.9% of the variance in quit attempts and 25.7% of the variance of smoking cessation one year later. The mean chi-square was 79.769, with 38 degrees of freedom. The attitude was adequately tapped by the three attitude items, in that their factor loadings exceeded 0.73, implying that 50% up to 93% of their variances were explained by the underlying factor.

**Figure 3:**
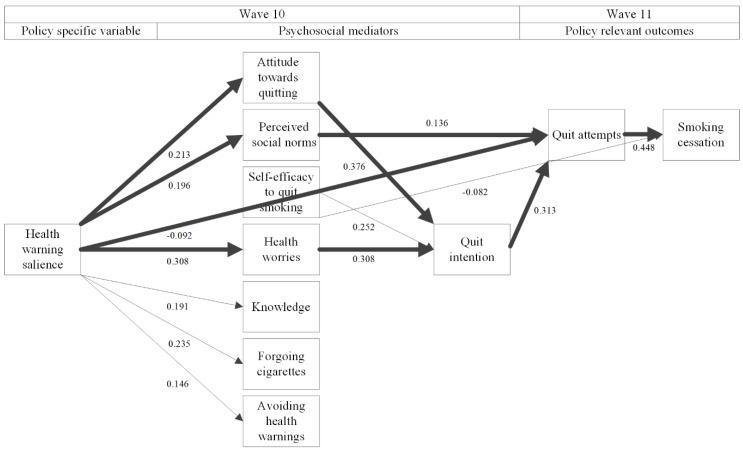
Structural equation model with standardized regression coefficients for the analysis of complete cases. Thick lines represent significant pathways between health warning salience and smoking cessation; thin lines represent significant associations that are not part of a significant mediational pathway; no lines between variables denote non-significant associations between them. All psychosocial mediators that are in the second column from the left were allowed to covary.

Health warning salience was positively associated with all psychosocial mediators, except for self-efficacy and quit intention. The associations between attitude towards quitting and health worries, on the one hand, and quit attempts on the other, were mediated by quit intention, while perceived social norms directly predicted quit attempts. Subsequently, quit attempts were positively associated with smoking cessation. The results of the analyses for complete cases (see Figure 3 and Table 5 for details) confirm these findings. There are significant pathways (1) from health warning salience, via perceived social norms, via quit attempts to smoking cessation (*p* = 0.023), (2) from health warning salience, via health worries, via quit intention, via quit attempts to smoking cessation (*p* < 0.001), and (3) from health warning salience, via attitude towards quitting, via quit intention to smoking cessation (*p* < 0.001). In addition, we now observed a significant pathway from health warning salience via quit attempts to smoking cessation (*p* = 0.009).

## 4. Discussion

This paper was the first to test the mediational pathways through which the new EU tobacco health warnings influence quit attempts and smoking cessation. We based our mediational model on the ITC conceptual model, which hypothesizes that policies influence quit attempts and smoking cessation via health warning salience as a policy specific variable and a set of eight potential psychosocial mediators [11]. The hypothesized model fitted the data very well, thus supporting the ITC conceptual model.

This study’s analysis revealed three significant pathways between PHW salience and smoking cessation. The pathway from health warning salience to quit intention to quit attempts [19,20], and to smoking cessation was mediated by two psychosocial mediators between health warning salience and quit intention. First, we found positive associations between health warning salience and attitude towards quitting, and between health warning salience and health worries. A study from Thailand also found a positive association between health warning salience and such attitude [39], and experimental studies showed that PHWs could be effective to elicit a negative smoking attitude [40]. Second, a positive association between health warning salience and health worries was found, in line with a previous study [10]. This might have been caused by the distressing messages as communicated by the health warnings [1]. The attitude and health worries were both associated with a stronger quit intention, in accordance with previous studies [10,15,16,19]. The third significant pathway ran from health warning salience—perceived social norms, quit attempts, and smoking cessation. Health warning salience might have been positively associated with stronger perceived social norms due to the accompanying (THWs): “Quit now—stay alive for close family and friends” [1]. Perceived social norms were associated with quit attempts at follow-up, as also found in a previous study [17], although sometimes, this association is mediated by quit intention [14,15,16,18].

Several pathways were not significant. First, in line with experimental studies [40], this study found that the efficacy messages “Quit now! Go to www.ikstopnu.nl., or call the quitline at 0800–1995 (free)” (1) were not enough to change self-efficacy levels. Second, in addition to inconclusive results on the direction and size of the association between health warning salience and quit intention in previous literature [4], we found no direct association between both variables. Furthermore, health warning salience was associated with three psychosocial mediators that were not directly or indirectly associated with quit intention, quit attempts, or smoking cessation. First, health warning salience was associated with more knowledge about the health risks of smoking, in line with other longitudinal observational studies [4]. The knowledge, as communicated by the current EU tobacco health warnings, may not have been enough to motivate smokers to quit smoking, contrary to another study [41], but sufficient research on this is lacking. Second, health warning salience was associated with increased self-reports that health warnings stopped smokers from having a cigarette when they were about to smoke one and avoiding health warnings. Similar associations were also found in experimental studies [40], and previous longitudinal observational studies [3,21,22,23,24]. Forgoing did not influence quit intention or attempts, in contrast to other observational studies that showed positive associations for forgoing [10,25]. These observational studies, however, showed weak associations and also included THWs without PHWs. In line with other population-based studies, avoidance of health warnings did not have negative effects on quit intention, quit attempts, or smoking cessation [25,26].

### 4.1. Strengths and Limitations

A major strength of this study is that due to the high number of respondents and the imputation of missing data, we had high statistical power. Another strength of this study is the use of a longitudinal design that is conducive for demonstrating the causality of the tested pathways between the psychosocial mediators and quit attempts and smoking cessation. However, the mediational model was partly cross-sectional, making it difficult to draw causal conclusions about the relationships between all outcomes, notably those between the policy-specific variable and the mediators, both of which were measured at the same wave. Nonetheless, since the construction of the mediational model tested in this study was based on the ITC conceptual model and behavior change theories, the results provide some support for the possible causal pathways from health warning salience to quit attempts. Relatedly, we could not examine the causal relationships between the psychosocial mediators. Our study has some limitations that should be acknowledged when interpreting the results. 

First, concerning the knowledge measures, we asked about some, but not all, of the health risks of smoking that were communicated by the EU tobacco health warnings. We did not ask about other health risks of smoking, such as reduced fertility, damage to teeth and gums, risks for children during pregnancy [1]. Therefore, we were unable to examine the full importance of knowledge about the health risks of smoking. Second, respondents who were lost to attrition differed from smokers who remained in the sample on 3 out of 16 variables. Although these variables were added as predictors in the analyses, thus providing some measure of controlling for the possible confounding, selection bias may still have occurred. Fourth, missing data always introduce some additional uncertainty, as evidenced by some small differences between the results based on multiple imputations and the complete case analysis. The similar results provide support for the validity of these mediational pathways. Finally, our findings were based on data from Dutch smokers who were exposed to EU tobacco health warnings. The results may, thus not be fully generalizable to other study populations or types of tobacco health warnings.

### 4.2. Implications

Our study suggests that the EU tobacco health warnings have the potential to influence quit attempts and smoking cessation via perceived social norms, attitudes towards quitting, and health worries. Our study results imply that tobacco health warnings can be used to increase smoking cessation, although future studies should examine how to make them even more effective. For instance, as our study revealed that perceived social norms are important in the process of smoking cessation, the PHWs might be more effective if the accompanying THWs would, e.g., convey a message about the percentage of family members who want their loved ones to quit smoking [42]. In addition, results show that health warning salience is an important variable to increase quit attempt. Therefore, it is important to increase health warning salience. This can be done by putting tobacco health warnings against a standardized background as previous studies showed that using ‘plain packaging’ may enhance health warning salience [43]. Furthermore, as self-efficacy in other studies [44] turns out to be an important predictor of smoking cessation, other behavior change methods that are often applied in smoking cessation counseling could be used to enhance self-efficacy levels of smokers, such as goal-setting, planning coping, or reattribution training [45]. Finally, as the results may not be fully generalizable to other study populations or types of tobacco health warnings, the working mechanisms of tobacco health warnings in other countries should be examined.

## 5. Conclusions

Our study provided a possible account of the working mechanisms of the EU tobacco health warnings. It revealed that these new health warnings exerted their influence on subsequent smoking cessation via three pathways. The first involved tobacco health warnings increasing smokers’ health worries, and the second involved stimulating positive attitudes towards quitting, both of which in turn had their influence on subsequent quitting behavior through increasing smokers’ quit intentions. The third pathway involved tobacco health warnings promoting positive social norms towards quitting, with a more direct impact on smokers (bypassing intention) by stimulating quit attempts directly, suggesting that the new tobacco health warnings may exert social influences that can motivate behavior change among smokers independent of their quit intentions.

## Figures and Tables

**Table 1:** Outcomes included in the analyses.

Outcome	Question Wording and Response Options
Policy specific variable (Wave 10)	
Health warning salience	1. In the last 30 days, how often, if at all, have you noticed photos on cigarette packages or on roll-your-own packs?—(1) Never, (2) Rarely, (3) Sometimes, (4) Often, (5) Very Often, or Don’t know (coded as missing)
	2. In the last 30 days, how often, if at all, have you read or looked closely at the photos on cigarette packages or on roll-your-own packs?—(1) Never, (2) Rarely, (3) Sometimes, (4) Often, (5) Very Often, or Don’t know (coded as missing)
Psychosocial mediators (Wave 10)	
Attitude towards quitting smoking	If you quit smoking within the next six months, this would be. 1. (1) Very foolish, (2) Foolish, (3) Not sensible and not foolish, (4) Wise, sensible, (5) Very wise and sensible or Don’t know (coded as missing)
	2. (1) Very disagreeable (2) Disagreeable (3) Not agreeable, but also not disagreeable, (4) Agreeable (5) Very agreeable (pleasant, gratifying), or Don’t know (coded as missing)
	3. (1) Very negative, (2) Negative, (3) Not positive and not negative, (4) Positive, (5) Very positive, or Don’t know (coded as missing)
Perceived social norms towards quitting	Thinking about the people who are important to you—how do you think most of them would feel about your quitting smoking within the next six months?—(1) Strongly disapprove, (2) Disapprove, (3) Neutral, (4) Approve, (5) Strongly approve, or Don’t know (coded as missing)
Self-efficacy to quit smoking	1. If you decided to give up smoking completely in the next six months, how sure are you that you would succeed?—(1) Not at all sure, (2) Slightly sure, (3) Moderately sure, (4) Very sure, (5) Extremely sure, or Don’t know (coded as missing)
	2. How easy or hard would it be for you to quit smoking if you wanted to?—(1) Extremely difficult, (2) Very difficult, (3) Moderately difficult, (4) Slightly difficult, (5) Not at all difficult, or Don’t know (coded as missing)
Health worries	How worried are you, if at all, that smoking will damage your health in the future?—(1) Not at all worried, (2) A little worried, (3) Moderately worried, (4) Very worried, or Don’t know (coded as missing)
Knowledge	Based on what you know or believe, does smoking cause… 1. Lung cancer—(0) No, (1) Yes, Don’t know (0) 2. Heart disease—(0) No, (1) Yes, Don’t know (0) 3. Impotence in male smokers—(0) No, (1) Yes, Don’t know (0) 4. Blindness—(0) No, (1) Yes, Don’t know (0) 5. Mouth and throat cancer—(0) No, (1) Yes, Don’t know (0) 6. Stroke—(0) No, (1) Yes, Don’t know (0)
Forgoing	In the last 30 days, have the warning labels stopped you from having a cigarette when you were about to smoke one?—(0) Never, (1) Once, (1) A few times, (1) Many times, or Don’t know (coded as missing)
Avoiding	In the last 30 days, have you made any effort to avoid looking at or thinking about the warning labels, such as covering them up, keeping them out of sight, using a cigarette case, avoiding certain warnings, or any other means?—No, Yes, or Don’t know (coded as missing)
Quit intention	Are you planning to quit smoking within the next 6 months?—(1) Very unlikely, (2) Unlikely, (3) Possibly, (4) Likely, (5) Very likely, or Don’t know (coded as missing)
Policy relevant outcomes (Wave 11)	
Quit attempts	Have you made any attempts (successful or not) to stop smoking in the last 12 months?—No, Yes
Smoking cessation	Smokers from Wave 10 who remained in the sample and who, in Wave 11, indicated to have quit smoking

**Table 2:** Sample characteristics of smokers in Wave 10 who remained in the sample in Wave 11, weighted by gender and age.

Measures	
**Control variables (Wave 10)**	
Gender (*n* = 1038)	
Male (%)	41.2
Female (%)	58.8
Age (*n* = 1038)	
15–24 (%)	9.9
25–39 (%)	20.4
40–45 (%)	31.9
55+ (%)	37.8
Education (*n* = 1018)	
Low (%)	24.3
Moderate (%)	44.1
High (%)	31.6
Level of nicotine dependence (mean, SD) (*n* = 1010)	2.06 (1.51)
Time in sample (mean, SD) (*n* = 1038)	4.75 (3.24)
Smoking frequency (*n* = 1038)	
Daily (%)	90
Non-daily (%)	10
**Policy specific variable (Wave 10)**	
Health warning salience (mean, SD) (*n* = 1012)	2.48 (1.09)
**Psychosocial mediators (Wave 10)**	
Attitude towards quitting (mean, SD) (*n* = 1018)	4.04 (0.80)
Perceived social norms (*n* = 986)	4.24 (0.81)
Self-efficacy (mean, SD) (*n* = 1008)	2.28 (1.05)
Health worries (mean, SD) (*n* = 920)	2.14 (0.76)
Knowledge (mean, SD) (*n* = 1038)	3.89 (1.93)
Forgoing (*n* = 979)	
No (%)	91.1
Yes (%)	8.9
Avoiding (*n* = 967)	
No (%)	79.7
Yes (%)	20.3
Quit intention (mean, SD) (*n* = 1003)	2.73 (1.22)
**Policy relevant outcomes (Wave 11)**	
Quit attempts (*n* = 898)	
No (%)	72.9
Yes (%)	27.1
Smoking cessation (*n* = 1023)	
No (%)	90.0
Yes (%)	10.0

**Table 3:** Spearman correlations between the policy-specific variables, psychosocial mediators, and policy-relevant outcomes, weighted by gender and age.

	1	2	3	4	5	6	7	8	9	10
**Policy specific variable**										
1. Health warning salience										
**Psychosocial mediators**										
2. Attitude towards quitting	0.165 ***									
3. Perceived social norms	0.182 ***	0.498 ***								
4. Self-efficacy	−0.025	−0.084 **	−0.184 ***							
5. Health worries	0.350 ***	0.368 ***	0.219 ***	0.041						
6. Knowledge	0.216 ***	0.260 ***	0.174 ***	0.044	0.298 ***					
7. Forgoing	0.206 ***	0.060	−0.060	0.070 *	0.191 ***	0.027				
8. Avoiding	0.163 ***	0.048	0.070 *	−0.074 *	0.166 ***	0.002	0.166 ***			
9. Quit intention	0.221 ***	0.343 ***	0.147 ***	0.294 ***	0.414 ***	0.176 ***	0.147 ***	0.060		
**Policy relevant outcomes**										
10. Quit attempts	0.040	0.164 ***	0.140 ***	0.113 **	0.217 ***	0.119 ***	0.040	0.031	0.373 ***	
11. Smoking cessation	−0.015	0.096 **	0.076 *	0.031	0.047	0.024	−0.015	−0.010	0.171 ***	0.476 ***

* *p* < 0.05, ** *p* < 0.01, *** *p* < 0.001.

**Table 4:** Standardized regression coefficients (β) of the structural equation model (after multiple imputations), weighted by age and gender ^a^.

	1	2	3	4	5	6	7	8	9	10
**Policy specific variable**										
1. Health warning salience										
**Psychosocial mediators**										
2. Attitude towards quitting	0.180 ***									
3. Perceived social norms	0.166 ***									
4. Self-efficacy	0.030									
5. Health worries	0.301 ***									
6. Knowledge	0.155 ***									
7. Forgoing	0.396 ***									
8. Avoiding	0.192 **									
9. Quit intention	0.042	0.340 ***	0.016	0.264 ***	0.304 ***	−0.070	0.172	−0.070		
**Policy relevant outcomes**										
10. Quit attempts	−0.068	−0.053	0.141 *	−0.030	0.046	0.044	0.008	0.010	0.336 **	
11. Smoking cessation	0.046	0.027	0.030	−0.087	−0.083	−0.044	−0.067	−0.029	0.036	0.452 *

* *p* < 0.05, ** *p* < 0.01, *** *p* < 0.001. ^a^ The columns contain the independent variables while the rows contain the dependent variables. The numbers in the columns refer to the corresponding variables with the same number in the rows.

**Table 5:** Standardized regression coefficients (β) of the structural equation model for the analysis on complete cases, weighted by age and gender ^a^.

	1	2	3	4	5	6	7	8	9	10
**Policy specific variable**										
1. Health warning salience										
**Psychosocial mediators**										
2. Attitude towards quitting	0.213 ***									
3. Perceived social norms	0.196 ***									
4. Self-efficacy	0.047									
5. Health worries	0.308 ***									
6. Knowledge	0.191 ***									
7. Forgoing	0.235 ***									
8. Avoiding	0.146 ***									
9. Quit intention	0.063	0.376 ***	−0.048	0.252 ***	0.308 ***	−0.040	0.059	−0.012		
**Policy relevant outcomes**										
10. Quit attempts	−0.092 **	−0.053	0.136 **	−0.004	0.076	0.042	0.002	0.007	0.313 **	
11. Smoking cessation	0.018	0.082	0.000	−0.082 *	−0.087	−0.043	−0.041	−0.017	−0.006	0.448 ***

* *p* < 0.05, ** *p* < 0.01, *** *p* < 0.001. ^a^ The columns contain the independent variables while the rows contain the dependent variables. The numbers in the columns refer to corresponding variables with the same numbers in the rows.
